# Association of multiple sclerosis with mortality in sepsis: a population-level analysis

**DOI:** 10.1186/s40560-022-00628-1

**Published:** 2022-07-25

**Authors:** Lavi Oud, John Garza

**Affiliations:** 1 Division of Pulmonary and Critical Care Medicine, Department of Internal Medicine, Texas Tech University Health Sciences Center at the Permian Basin, 701 W. 5th Street, Odessa, TX 79763 USA; 2 Texas Tech University Health Sciences Center at the Permian Basin, 701 W. 5th Street, Odessa, TX 79763 USA; 3 Department of Mathematics, The University of Texas of the Permian Basin, 4901 E. University Blvd, Odessa, TX 79762 USA

**Keywords:** Multiple sclerosis, Sepsis, Septic shock, Intensive care unit, Mortality

## Abstract

**Background:**

Multiple sclerosis (MS) is associated with increased risk of sepsis and higher sepsis-related mortality, compared to the general population. However, the evidence on the prognostic impact of MS in sepsis has been scarce. We aimed to evaluate the population-level association of MS with short-term mortality in sepsis.

**Methods:**

We performed a retrospective population-based cohort study using a statewide data set to identify hospitalizations aged ≥ 18 years in Texas with sepsis, with and without MS during 2010–2017. Multilevel logistic models were fit to estimate the association of MS with short-term mortality among all sepsis hospitalizations, and for sensitivity analyses among hospitalizations with septic shock and those admitted to ICU.

**Results:**

Among 283,025 sepsis hospitalizations, 1687 (0.6%) had MS. Compared to sepsis hospitalizations without MS, those with MS were younger (aged ≥ 65 years 35.0% *v*s 56.8%), less commonly racial/ethnic minority (36.2% vs 48.1%), and had lower mean Deyo comorbidity index (1.6 vs 2.7). The rates of septic shock and ICU admission were similar for sepsis hospitalizations with and without MS (58.7% vs 59.6% and 46.7% vs 46.0%, respectively). The unadjusted short-term mortality among sepsis hospitalizations with and without MS for the whole cohort, among those with septic shock, and among ICU admissions were 20.2% vs 31.3%, 25.6% vs 40.0%, and 24.0% vs 34.8%, respectively. On adjusted analyses, MS was associated with 17% lower odds of short-term mortality (adjusted odds ratio [aOR] 0.828 [95% CI 0.723–0.947]). Similar findings were observed on sensitivity analyses of patients with septic shock (aOR 0.764 [95% CI 0.651–0.896]), but MS was not associated with mortality among sepsis hospitalizations admitted to ICU (aOR 0.914 [95% CI 0.759–1.101]).

**Conclusions:**

MS was associated with lower short-term mortality among septic patients, with findings consistent among the subset with septic shock. Among septic patients admitted to ICU, MS was not associated with mortality.

**Supplementary Information:**

The online version contains supplementary material available at 10.1186/s40560-022-00628-1.

## Background

Multiple sclerosis (MS) is the most common autoimmune inflammatory demyelinating disease of the central nervous system, affecting approximately 0.3% of the United States population [1]. Despite marked advances in the care of patients with MS, affected patients continue to have reduced life expectancy, compared to the general population [2].

Patients with MS face an increased risk of infectious complications, which may in turn progress to sepsis, due to disease-related physiological changes [3, 4] and use of immunosuppressive therapy [5], though the magnitude of the latter risk remains unsettled [6, 7].

Sepsis is in turn a major cause of death among patients with MS [8], with affected patients having an increased risk of sepsis-related mortality compared to those without MS [9]. The higher risk of sepsis-related mortality in patients with MS compared to the general population may reflect their increased risk of sepsis, greater case fatality among septic patients, or both. Distinction between these factors is important, as each would lead to different interventions. However, while greater risk of sepsis among patients with MS is well-documented, being nearly 6-times higher, compared to those without MS [10], the evidence base on the prognostic impact of MS in sepsis has been scarce.

Only one study to date has examined, to our knowledge, the prognostic association of MS with short-term mortality in sepsis. In a recent report on the prognostic impact of autoimmune diseases in sepsis by Sheth et al., 30-day mortality was markedly lower among these patients as a group compared to those without autoimmune disease, but was not different statistically in the subgroups of individual disease, except among those with MS [11]. However, the interpretation and external validity of this study’s findings are limited by its single-center design, small number of patients in the MS subgroup (*n* = 64), restriction to ICU patients, and lack of data on patient characteristics and mortality rate of the MS subgroup [11].

A better understanding of the prognostic impact of MS in sepsis can inform clinicians’ decision-making, future interventional efforts to improve sepsis outcomes, and provide benchmarking for performance improvement. Here, we report a population-based study of hospitalizations with sepsis to examine the association of MS with short-term mortality.

## Materials and methods

This was a retrospective, population-based cohort study. The study was determined to be exempt from formal review by the Texas Tech Health Sciences Center’s Institutional Review Board, because we used a publicly available, de-identified data set. The reporting of the study findings followed the STROBE guidelines on reporting observational studies in epidemiology [12].

### Data sources and study population

We used the Texas Inpatient Public Use Data File (TIPUDF) to identify the target population. In brief, the TIPUDF is an administrative data set maintained by the Texas Department of State Health Services [13] and includes inpatient discharge data from state-licensed, non-federal hospitals, and captures approximately 97% of all hospital discharges in the state.

Our primary cohort consisted of hospitalizations aged ≥ 18 years with a diagnosis of sepsis during the years 2014–2017. We excluded hospitalizations with missing data on hospital disposition. We identified hospitalizations with sepsis based on the presence of the International Classification of Diseases, Ninth and Tenth Revisions, Clinical Modification (ICD-9-CM and ICD-10-CM, respectively) codes for severe sepsis (995.92, R65.20) and septic shock (785.52, R65.21) under the principal or secondary diagnosis fields. This ICD code-based definition of sepsis is aligned with the framework of Sepsis-3 [14] and has been used in contemporary studies of sepsis in administrative data [15–17]. Hospitalizations with ICU admissions were identified based on unit-specific revenue codes for an intensive care unit or a coronary care unit.

### Exposure and outcome

The primary exposure was a diagnosis of MS. We identified hospitalizations with MS, based on the presence of ICD-9-CM and ICD-10-CM codes 340 and G35, respectively, in the principal or secondary diagnosis fields [18, 19]. The primary outcome was short-term mortality, defined as in-hospital death or discharge to hospice. We have included discharge to hospice, since this is an increasingly common end-of-life destination in sepsis; therefore, focusing only on in-hospital mortality can produce misleading estimates and for this reason this composite outcome is increasingly used in epidemiological studies of sepsis [15].

### Risk-adjustment covariates

Risk-adjustment covariates were selected a priori based on biological and clinical plausibility and existing literature [20–22] and included patients’ demographics (age, gender, race/ethnicity, primary health insurance), major comorbidities (based on the Deyo modification of the Charlson Comorbidity Index [23, 24]), sites of infection, hospitals’ teaching status, and year of admission. Severity of illness was characterized using ICD codes for organ dysfunction [25]. Procedure use was identified using ICD-9 and ICD-10 procedure codes for mechanical ventilation, hemodialysis, and blood transfusion. The ICD-9-CM and ICD-10-CM codes used to identify sites of infection, organ dysfunction, and procedures are detailed in Additional files 1, 2, 3.

### Statistical analysis

We summarized categorical variables as frequencies and percentages, while continuous variables were reported as mean and standard deviation. The chi-square test was used for group comparison involving categorical variables, while the *t* test was used for comparison of continuous variables.

We used multilevel logistic regression to estimate the association of the independent variable MS with the dependent variable short-term mortality, among sepsis hospitalizations. The covariates entered into the multivariable model included all those described for risk-adjustment, as well as MS, and with individual hospitals entered as random intercepts to account for clustering of hospitalizations within hospitals. Multicollinearity was assessed using variance inflation factors. We report the model’s findings as adjusted odds ratios (aOR) and 95% confidence intervals (95% CI).

#### Subgroup analyses

Exploratory analyses were performed to examine the consistency of the association between MS and short-term mortality among a priori selected subgroups, including age, gender, race/ethnicity, Deyo comorbidity index, and the number of organ dysfunctions. The primary analysis approach was used for subgroup analyses.

#### Sensitivity analyses

We probed the robustness of the observed association between MS and short-term mortality with two additional analyses, restricted to those more severely ill, including those with septic shock and sepsis hospitalizations admitted to ICU. The primary analysis approach was used for sensitivity analyses.

The State of Texas masks gender data of hospitalizations with a diagnosis of HIV infection, and of those with ethanol or drug abuse. Gender data were missing nonrandomly in 8.8% of hospitalizations in our cohort, precluding imputation of missing values. Using the primary analysis approach we examined the sensitivity of the association between MS and short-term mortality to missing gender data using missing gender as indicator variable for the whole cohort and for sensitivity analyses. In this article, we present the results of our analyses using data restricted to hospitalizations with gender data.

Data management was performed using Microsoft Excel (Microsoft, Redmond, Washington) and statistical analyses were performed with R 4.0.5 (R Foundation for Statistical Computing, Vienna, Austria). The R code supporting these analyses is provided in Additional file 4. A two-sided *p* value < 0.05 was considered statistically significant.

## Results

### Cohort characteristics

Among 283,025 hospitalizations with sepsis, meeting inclusion criteria, 1,687 (0.6%) had MS.

The characteristics of sepsis hospitalizations with and without MS for the whole cohort are detailed in Table 1. Compared to sepsis hospitalizations without MS, those with MS were younger (aged ≥ 65 years 35.0% vs 56.8%), less commonly racial/ethnic minority (36.2% vs 48.1%), had lower burden of comorbidities (mean Deyo comorbidity index 1.6 vs 2.7), lower mean number of organ dysfunctions (2.3 vs 2.7) and lower use of mechanical ventilation (29.9% vs 35.0%). Sepsis hospitalizations with MS and without MS had similar rates of admission to ICU (46.7% vs 46.0%) and septic shock (58.7% vs 59.6%).

**Table 1:** Characteristics of sepsis hospitalizations with and without multiple sclerosis

Variables	Multiple sclerosis^a^	Non-multiple sclerosis^a^	*p* value
	*n* = 1687	*n* = 281,338	
Age, years			< 0.0001
18–44	227 (13.5)	30,630 (10.9)	
45–64	869 (51.5)	90,844 (32.3)	
≥ 65	591 (35.0)	159,864 (56.8)	
Gender^b^			
Female	1119 (68.3)	128,993 (50.3)	< 0.0001
Race/ethnicity			< 0.0001
White	1077 (63.8)	146,142 (51.9)	
Hispanic	247 (14.6)	75,235 (26.7)	
Black	257 (15.2)	36,783 (13.1)	
Other	106 (6.3)	23,116 (8.2)	
Health insurance			< 0.0001
Private	507 (30.0)	91,957 (32.7)	
Medicare	1001 (59.3)	140,174 (49.8)	
Medicaid	133 (7.9)	20,953 (7.4)	
Uninsured	34 (2.0)	24,317 (8.6)	
Other	12 (0.7)	3592 (1.3)	
Deyo comorbidity index^c^	1.6 (1.7)	2.7 (2.4)	< 0.0001
Selected comorbidities			
Chronic lung disease	343 (20.3)	75,230 (26.7)	< 0.0001
Congestive heart failure	242 (14.3)	87,544 (31.1)	< 0.0001
Renal disease	265 (15.7)	94,216 (33.5)	< 0.0001
Diabetes	436 (25.8)	112,636 (40.0)	< 0.0001
Malignancy	84 (5.0)	40,417 (14.4)	< 0.0001
Liver disease	53 (3.1)	33,938 (12.1)	< 0.0001
Site of infection			
Respiratory	535 (31.7)	105,601 (37.5)	< 0.0001
Urinary	1078 (63.9)	103,365 (36.7)	< 0.0001
Abdominal	135 (8.0)	34,813 (12.4)	< 0.0001
Skin and soft tissue	114 (6.8)	27,964 (9.9)	< 0.0001
Device-related	78 (4.6)	6915 (2.5)	< 0.0001
Other^d^	106 (6.3)	15,383 (5.5)	0.1509
Septic Shock	990 (58.7)	167,808 (59.6)	0.4526
Number of organ dysfunctions^c^	2.3 (1.4)	2.7 (1.5)	< 0.0001
Type of organ dysfunctions			
Respiratory	826 (49.0)	152,684 (54.3)	< 0.0001
Cardiovascular	1050 (62.2)	180,694 (64.2)	0.0876
Renal	781 (46.3)	172,187 (61.2)	< 0.0001
Hepatic	56 (3.3)	24,905 (8.9)	< 0.0001
Hematological	217 (12.9)	58,772 (20.9)	< 0.0001
Neurological	548 (32.5)	778,873 (27.7)	< 0.0001
ICU admission	788 (46.7)	129,370 (46.0)	0.5652
Mechanical ventilation	505 (29.9)	98,361 (35.0)	< 0.0001
Hemodialysis	63 (3.7)	35,812 (12.7)	< 0.0001
Blood transfusion	257 (15.2)	55,891 (19.9)	< 0.0001
Teaching hospital	401 (23.8)	78,549 (27.9)	< 0.0001
Hospital disposition			
In-hospital death	224 (13.3)	65,213 (23.2)	< 0.0001
Hospice	117 (6.9)	22,921 (8.1)	0.0716
Home	528 (31.3)	100,604 (35.8)	0.0001
Another acute care hospital	47 (2.8)	10,244 (3.6)	0.0785
Post-acute care facility^e^	764 (45.3)	80,254 (28.5)	< 0.0001
Leave against medical advise	7 (0.4)	2102 (0.7)	0.1401

^a^The parenthesized figures represent percents, except for Deyo comorbidity index and number of organ dysfunctions; Percentage figures may not add to 100 due to rounding

^b^Gender was missing for 51 (3.0%) hospitalizations with multiple sclerosis arthritis and for 24,943 (8.9%) hospitalizations. without multiple sclerosis; the percent figures for gender in each column refer to that column's denominator for gender

^c^Mean (standard deviation [SD])

^d^Other sites of infection include endocarditis, blood, genital, bone and joint, and the central nervous system

^e^Post-acute care facilities include: long-term care hospitals, inpatient rehabilitation, skilled nursing facilities, and nursing homes

### The impact of MS on short-term mortality

The details of hospital disposition of cohort hospitalizations are provided in Table 1. Sepsis hospitalizations with MS had markedly lower unadjusted short-term mortality compared to those without MS (20.2% vs 31.3%, respectively; *p* < 0.0001). Similarly, unadjusted short-term mortality was substantially lower among sepsis hospitalizations with MS than those without MS among those with septic shock (25.6% vs 40.0%; *p* < 0.0001) and those admitted to ICU (24.0% vs 34.8%; *p* < 0.0001). The reduced risk of short-term mortality among sepsis hospitalizations with MS remained on adjusted analyses for the whole cohort, with 17% lower odds of death (aOR 0.828 [95% CI 0.723–0.947]; *p* = 0.0061).

**Fig. 1:**
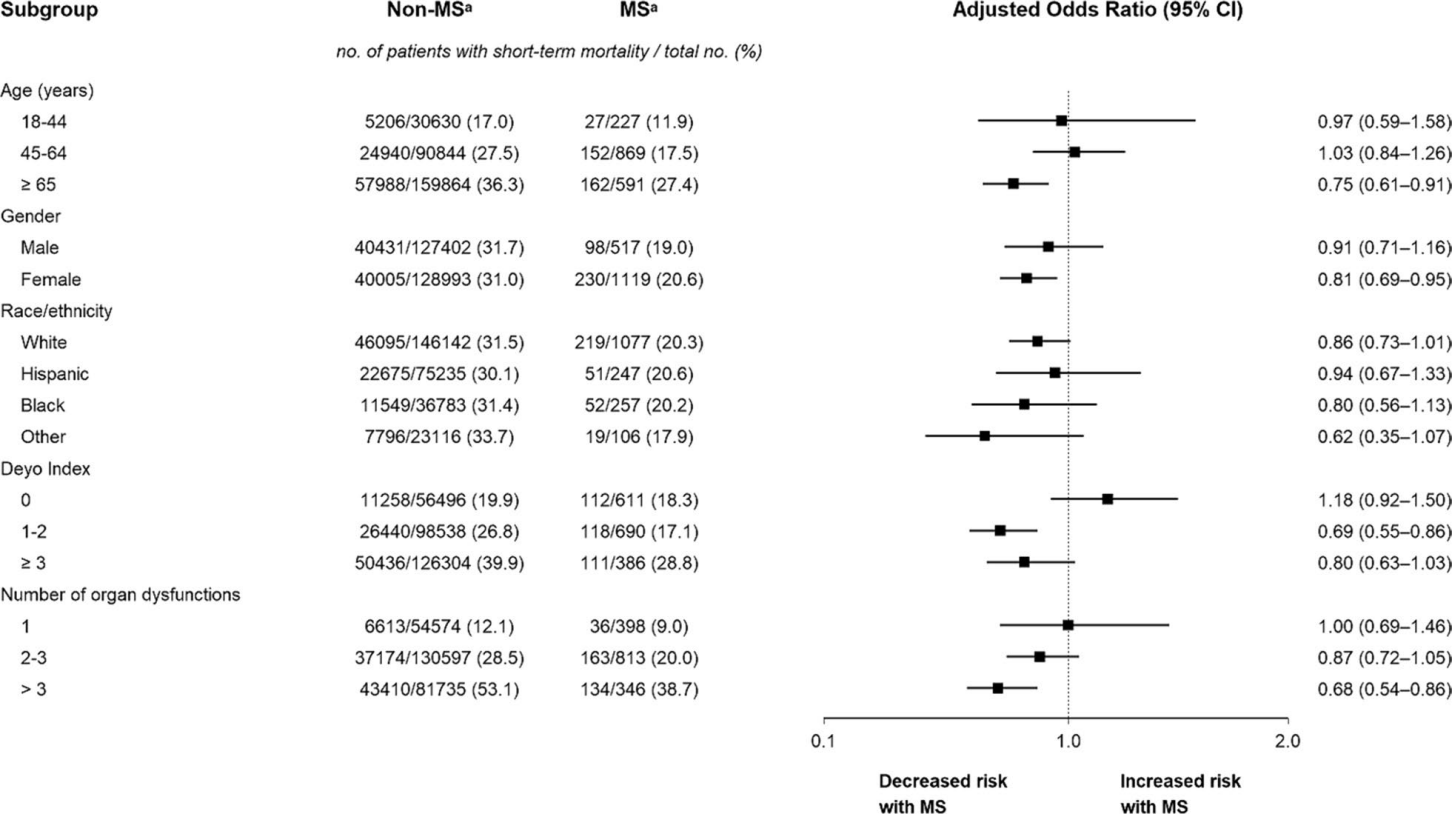
Subgroup analyses of short-term mortality of sepsis hospitalizations with and without multiple sclerosis. Analyses were done using multilevel logistic regression. The odds ratios and 95% confidence intervals have not been adjusted for multiplicity and should not be used to infer definite effects. ^a^MS indicates multiple sclerosis

On subgroup analyses, unadjusted short-term mortality was consistently lower among patients with MS compared to those without MS across all examined strata of sepsis hospitalizations. The point estimates of the odds of short-term mortality on adjusted analyses were generally consistent with the primary analysis. Notably, on adjusted analyses, the odds of short-term mortality tended to be especially lower among sepsis hospitalizations with MS who were older and those with higher number of organ dysfunctions (Fig. 1).

Sensitivity analyses showed similar findings of the association of MS with short-term mortality as the primary model among sepsis hospitalizations with septic shock (aOR 0.764 [95% CI 0.651–0.896]; *p* = 0.0010). However, MS was not associated with short-term mortality among those admitted to ICU (aOR 0.914 [95% CI 0.759–1.101]; *p* = 0.3457). The alternative modeling approach for missing gender has produced similar findings for the whole cohort, as well as for sepsis hospitalizations with septic shock and those admitted to ICU (see Additional files 5, 6, 7).

## Discussion

### Key findings

In this population-based study, we found that short-term mortality was substantially lower among septic patients with MS than among those without MS. This lower risk of short-term mortality was also present, following adjustment for confounders, among patients with MS and septic shock, but not among septic patients managed in the ICU, where MS was not associated with mortality.

### Relationship to prior studies

The “protective” association of MS with short-term mortality among septic patients in our study’s cohort extends the findings of the single-center study reported by Sheth et al. [11] to a population level. However, the findings of that study cannot be further compared to our cohort, because patient characteristics and the mortality rate of the sepsis subgroup with MS were not reported [11].

Septic patients with MS in our cohort were younger than those without MS, less commonly racial or ethnic minority, and had lower burden of chronic illness, as well as lower severity of illness, all of which would be expected to contribute to lower lethality of sepsis, compared to the general population. However, the lower risk of short-term mortality among septic patients with MS for the whole cohort, as well as in the subset with septic shock and generally in the point estimates of the subgroup analysis remained following adjustment for group imbalances. Our findings suggest that, when compared to the general population, the higher risk of sepsis-related mortality in patients with MS [8] reflects the increased risk of sepsis among the latter, rather than higher case fatality of septic patients.

Our finding of lack of association of MS with short-term mortality among sepsis hospitalizations admitted to ICU on adjusted analysis contrasts, unexpectedly, with the study by Sheth et al., though their cohort was confined to septic patients managed in the ICU [11]. However, in a recent multicenter study by Anesi et al. [26], the risk of hospital mortality on adjusted analyses among septic patients with an equal expectation for ICU or ward admission was higher among the former. The investigators hypothesized that septic patients admitted to ICU may have been exposed to greater use of harmful intervention, higher frequency of complications, or different end-of-life care processes [26]. While not directly reflecting our cohort, we speculate that these types of ICU-level exposures of septic patients with and without MS may have had greater adverse effect on the former and have eliminated their differences of adjusted short-term outcomes. Importantly, it may be that the potential harms associated with ICU admissions with sepsis hypothesized by Anesi et al. [26] have not been as prominent in the cohort described by Sheth et al., as it involved patients managed at a single high-performing academic center [11], which may not be representative of critical care practices across heterogenous health care systems. In the interim, our findings show that care escalation to ICU among septic patients with MS does not indicate worse short-term outcome compared to those in the general population.

The mechanisms underlying the lower risk of short-term mortality associated with MS among septic patients are unclear. Dysregulated immune response to infection, including imbalance of pro- and anti-inflammatory cytokines [27] is considered a key driver of sepsis manifestations [28], and the aberrant cytokine signatures among patients with autoimmune diseases, including MS, include some of those involved in the pathophysiology of sepsis [29, 30]. And may have affected the response to infection and subsequent sepsis among the latter [11].

In the recent single-center study by Sheth et al., noted earlier, autoimmune diseases were associated as a group with lower risk-adjusted 30-day mortality (aOR 0.73 [95% CI 0.57–0.93]) [11], though the favorable prognostic association was generally not statistically significant for individual diseases. However, when analyses in that study were stratified by levels of cytokine expression of individual autoimmune diseases, those with overexpression of Interleukin-12 (IL-12) and interferon-gamma (IFN-γ) were associated, as a group, with lower risk of death, with similar trends among those diseases with underexpression of Interleukin-4 (IL-4) and Interleukin-10 (IL-10) [11]. A similar pattern of cytokine expression was reported in patients with MS, with both IL-12 [31, 32] and IFN-γ [31–34] being overexpressed, while IL-4 and IL-10 are underexpressed [32, 34]. These “protective” associations between pre-sepsis immune dysregulation and mortality are supported by studies showing that sepsis impairs production of IL-12 [35] and IFN-γ [36], with preclinical studies showing that therapies that increase the expression of both can improve sepsis survival [37]. In addition, the immunosuppressive effect of sepsis is augmented by release of IL-4 and IL-10 [36], both of which were shown to reduce expression of pro-inflammatory cytokines. The authors have hypothesized that patients with pre-sepsis over-expression of specific cytokines may be better suited to survive sepsis-induced impairment in immune function [11]. If correct, this hypothesis may explain the findings of the present study.

However, notwithstanding the compelling prognostic associations noted in the study by Sheth et al. [11] and the cytokine profiles reported in patients with MS, there have not been, to our knowledge, direct comparative studies on the immune responses of septic patients with and without MS. The prognostic impact of immunosuppressive therapy in sepsis among patients with MS has not been reported, to our knowledge. However, in the study by Sheth el, immunosuppressive therapy was not associated with mortality in septic patients with autoimmune diseases and, specifically, did not appear to mediate the outcomes of these septic patients as a group (which included MS) [11].

Finally, the factors underlying our findings of comparatively greater “protective” association of MS with short-term mortality among older patients and those with higher number of organ dysfunctions on point estimates of adjusted subgroup analyses are unclear. We speculate that the effects of accelerated immunosenescence [38] among patients with MS on their response to infection may have been more prominent and “protective” in older patients, while the subset of septic MS patients with greater number of organ dysfunctions may have had much lower severity of individual organ dysfunctions than those without MS, as compared to those with lower number of organ dysfunctions. However, because subgroup estimates represent exploratory analyses, further confirmatory studies are needed prior to mechanistic investigations of the sources of these observations.

### Study implications

The focus of our study differs from that of other investigations designed to identify patient groups (or specific conditions) associated with different mortality outcomes in sepsis compared to the general population. Such studies generally describe patient groups with higher mortality than cohorts’ baseline. A key long-term goal based on the findings of these studies is to determine the mechanisms underlying the observed outcome differences and, importantly, possibly identify modifiable mechanisms that can serve as targets for interventions to reduce outcome disparities of the affected patient group, ideally bringing its outcomes to the same level as those in the general population.

We chose an opposite approach, focusing on a disease with known pre-sepsis immune dysfunction, which places affected patients at an increased risk of sepsis [10] and can be expected to be associated with higher risk of death once sepsis has developed (as has been reported, for instance, in patients infected with the human immunodeficiency virus or those with cancer), but where patients were actually found to have, unexpectedly, lower short-term mortality than septic patients in the general population [11]. This type of findings can provide potential opportunities to gain broader and deeper insights on the pathogenesis of sepsis and its impact on patient outcomes. Our study was thus designed to determine whether the findings by Sheth et al. [11] for septic patients with MS are robust at a population level, rather than representing a potential chance occurrence at a single center with only a few dozen patients.

Because differences in host response to infection between septic patients with and without MS appear to represent a plausible explanation for our findings, further studies are warranted to characterize the comparative sepsis-associated changes across immune function domains in patients with and without MS, to provide mechanistic insights that may guide identification of potential interventions to improve sepsis outcomes in the general population. Given the current lack of effective interventions to modulate the dysregulated host response to infection, which is considered the driver of sepsis and its lethal outcomes, we consider this line of investigation worth pursuing. Recent reports characterizing sepsis endotypes through mechanistic signatures of gene expression of immune effectors to predict sepsis severity [39] may inform such studies.

### Strengths and limitations

Our study has relevant strengths and limitations. In terms of strengths, the present study evaluates a relatively little-examined and important research question. The use of a statewide, all-payer, high-quality data set of consecutive hospitalizations allowed transcending local variation in case mix and practice patterns.

This study has, however, important limitations, mostly related to the retrospective design and use of administrative data. First, although the ICD codes for MS in the present report were used in prior epidemiological studies [18, 19], we cannot exclude misclassification between groups. However, misclassification of MS hospitalizations would be expected to blur the differences between groups and thus diminish outcome differences between septic patients with and without MS, leading to possible underestimation of the magnitude of the better outcomes observed among the former. Second, our data set did not include information of the duration and type of MS, its activity level, or details on immunomodulating therapy. In addition, the TIPUDF data did not include information on processes of care and their timeliness, which may have differed between septic patients with and without MS. Thus, we cannot exclude residual confounding in our models. Third, our study did not directly ascertain mortality of septic patients that occurred after hospital discharge. Last, the generalizability of our findings to other countries and regions, is unknown.

## Conclusions

MS was associated with lower short-term mortality among septic patients. This favorable prognostic association was generally consistent on subgroup analyses, and among patients with septic shock, but not among septic patients admitted to ICU. Future studies are needed to determine the mechanisms underlying these observations, to inform efforts to improve sepsis outcomes.

## Supplementary Information

**Additional file 1:** International Classification of Diseases*,* Ninth and Tenth Revisions*,* Clinical Modification (ICD-9-CM and ICD-10-CM) codes used to identify sites of infection.

**Additional file 2:** International Classification of Diseases*,* Ninth and Tenth Revisions*,* Clinical Modification (ICD-9-CM and ICD-10-CM) codes used to identify organ dysfunctions.

**Additional file 3:** International Classification of Diseases*,* Ninth and Tenth Revisions*,* Clinical Modification (ICD-9-CM and ICD-10-CM) codes used to identify procedures.

**Additional file 4**: R code used for study modeling.

**Additional file 5:** Multilevel logistic regression for the association of multiple sclerosis with short-term mortality for the whole cohort: alternative modeling for the impact of missing gender data.

**Additional file 6:** Multilevel logistic regression for the association of multiple sclerosis with short-term mortality among hospitalizations with septic shock: alternative modeling for the impact of missing gender data.

**Additional file 7:** Multilevel logistic regression for the association of multiple sclerosis with short-term mortality among hospitalizations admitted to ICU: alternative modeling for the impact of missing gender data.

## Data Availability

The data set supporting the conclusions of this article is available through the Texas Department of State Health Services, Center for Health Statistics, Austin, Texas, at http://www.dshs.state.tx.us/thcic/hospitals/Inpatientpudf.shtm.

## Abbreviations

ICD-9-CM: International Classification of Diseases, Ninth Revision, Clinical Modification
ICD-10-CM: International Classification of Diseases, Tenth Revision, Clinical Modification
MS: Multiple sclerosis
TIPUDF: Texas inpatient public use data file
