# Supplementary material for: Association of multiple sclerosis with mortality in sepsis: a population-level analysis
Source: J Intensive Care. 2022 Jul 25;10:36. doi: 10.1186/s40560-022-00628-1 (PMC9310428; doi:10.1186/s40560-022-00628-1)
Supplement: Supplementary file 1 — Additional file 1: International Classification of Diseases, Ninth and Tenth Revisions, Clinical Modification (ICD-9-CM and ICD-10-CM) codes used to identify sites of infection. [file 40560_2022_628_MOESM1_ESM.docx]

**eTable 1. International Classification of Diseases*,* Ninth and Tenth Revisions*,* Clinical Modification (ICD-9-CM and ICD-10-CM) codes used to identify sites of infection.**

**Site of infection ICD-9-CM codes ICD-10-CM codes**

Respiratory 481x, 482x, 483x, 484x, 485x, 486x, 4870x, 4871x J13x, J14x, J15x, J16x, J17x, J18x, B250, A3791,

48801, 48802, 48811, 48812, 48881, 48882, 510x A221, B440, J860, J869, J85x

513x

Urinary 590x, 5990x N110, N118, N10, N151, N2884, N2885, N2886

N12, N16, N159, N30x, N390

Abdominal 003x, 008x, 540x, 541x, 542x, 5304x, 53086, 56201 A02x, A03x, A05x, A08x, K35x, K36x, K37x, K65x,

56203, 56211, 56213, 566x, 567x, 5695x, 56983 K67x, K68x, K223, K9432, K57x, K751, K61x, K630

5720x, 5750x, 5311x, 5312x, 5315x, 5316x, 5321x K631, K750, K810, K812, K251, K252, K253, K255

5322x, 5325x, 5326x, 5331x, 5332x, 5335x, 5336x K256, K257, K259, K261, K262, K263, K265, K266

5341x, 5342x, 5345x, 5346x K267, K281, K282, K285, K286, K271, K272, K275

K276

Skin and soft tissues 6751x, 680x, 682x, 686x, 9985 O91x, L02x, K122, L03x, L08x, K6811

Blood 7907x, 5721x, 6733 R7881

Endocarditis 11281, 421x, 42490, 42491, 11504, 11594, 03642 B376, I330, I39, I339, I38, I39, B394, B399, A5483

09884

Genital 615x, 6340x, 6350x, 636x, 6370x, 6380x, 6390x N71x, O045, O0487, N710, N711, N719, O035, O0387

6466x, 6584x O030, O0337, O035, O0O45, O070, O0737, O080,

O0882

Central nervous system 320x, 322x, 324x, 325x G00x, G01, G03x, G042, G06x

Bone and joint 7710x, 730x M0Ox, M86x

Device-related 9966x T8579XA, T80211x, T80212x, T80218x, T80219x
