# Supplementary material for: Association of multiple sclerosis with mortality in sepsis: a population-level analysis
Source: J Intensive Care. 2022 Jul 25;10:36. doi: 10.1186/s40560-022-00628-1 (PMC9310428; doi:10.1186/s40560-022-00628-1)
Supplement: Supplementary file 2 — Additional file 2: International Classification of Diseases, Ninth and Tenth Revisions, Clinical Modification (ICD-9-CM and ICD-10-CM) codes used to identify organ dysfunctions. [file 40560_2022_628_MOESM2_ESM.docx]

**eTable 2. International Classification of Diseases*,* Ninth and Tenth Revisions*,* Clinical Modification (ICD-9-CM and ICD-10-CM) codes used to identify organ dysfunctions.**

**Organ dysfunction ICD-9-CM codes ICD-10-CM codes**

Respiratory 51881, 51882, 51884, 78609, 7991 J9600, J9601, J9602, J9620, J9621, J9622, J80, R0603,

R0600, R0609, R092

Cardiovascular 4580, 785x, 4588x, 4589x, 7963 I9589, I959, I951, R579, R570, R6521, R571, R578, R031

Renal 580x, 584x N170, N171, N172, N178, N179

Hepatic 570x, 5722x, 5723x K7200, K762, K7290, K7291, K763

Hematological 2862x, 2866x, 2869x, 2873x, 2874x, 2875x D65, D688, D689, D6959, D696

Neurological 293x, 3481x, 3483x, 78001, 78009, 8914 F05, F062, F060, F0630, F064, F061, F068, G931, G9340,

G9341, G9349, R4020, R400, R401
