# Supplementary material for: Association of multiple sclerosis with mortality in sepsis: a population-level analysis
Source: J Intensive Care. 2022 Jul 25;10:36. doi: 10.1186/s40560-022-00628-1 (PMC9310428; doi:10.1186/s40560-022-00628-1)
Supplement: Supplementary file 3 — Additional file 3: International Classification of Diseases, Ninth and Tenth Revisions, Clinical Modification (ICD-9-CM and ICD-10-CM) codes used to identify procedures. [file 40560_2022_628_MOESM3_ESM.docx]

**eTable 3. International Classification of Diseases*,* Ninth and Tenth Revisions*,* Clinical Modification (ICD-9-CM and ICD-10-CM) codes used to identify procedures.**

**Procedure ICD-9-CM codes ICD-10-CM codes**

Mechanical ventilation 9670, 9671, 9672 5A1935Z, 5A1945Z, 5A1955Z

Hemodialysis 3895, 3995, V4511, V560, V561 Z4901, 5A1D00Z, 5A1D60Z, 5A1D70Z, 5A1D80Z,

5A1D90Z

Blood transfusion 9900, 9901, 9902, 9903, 9904, 9905 30233N1, 30243N1, 30253K1,30243K1

9906, 9907, 9908, 9909, V582 30253L1, 30243L1, 30233R1, 30243R1,

30240N0,  30240N1, 30230N0,   30230N1,  30240K0,

30240K1, 30240L0, 30240L1,  30240M0, 30240M1,

30230L0,  30230L1, 30230M0,  30230M1,  30240R0,

30240R1, 30230R0, 30230R1
