# Supplementary material for: Association of multiple sclerosis with mortality in sepsis: a population-level analysis
Source: J Intensive Care. 2022 Jul 25;10:36. doi: 10.1186/s40560-022-00628-1 (PMC9310428; doi:10.1186/s40560-022-00628-1)
Supplement: Supplementary file 4 — Additional file 4: R code used for study modeling. [file 40560_2022_628_MOESM4_ESM.docx]

**R code used for study modeling**

# Multilevel Logistic Regression

model <-

glmer(

formula = m_h ~

age + sex + race_ethnicity + insurance + deyoindex +

deyolung + deyocvd + deyockd + deyochf + dm +

chrliver + malign + year + procimvall + oftotal +

prochd + procbld + ms + teaching + infection +

(1|thcic_id),

data = M,

family = binomial,

control = glmerControl(

optimizer = 'nloptwrap',

optCtrl = list(maxfun = 1e6)),

start = NULL,

verbose = 0L,

nAGQ = 0,

contrasts = NULL,

devFunOnly = FALSE)

# Define model summary

sepsis_ms <- summary(model)

# Extract model coefficients

estimates_ms <- sepsis_ms$coefficients[, 1]

std_ms <- sepsis_ms$coefficients[, 2]

p_value_ms <- sepsis_ms$coefficients[, 4]

# Compute 95% confidence intervals

M_ms <-

tibble(

name = rownames(sepsis_ms$coefficients),

L = round(exp(estimates_ms - 1.96 * std_ms), digits = 4),

OR = round(exp(estimates_ms), digits = 4),

U = round(exp(estimates_ms + 1.96 * std_ms), digits = 4),

p_value = round(p_value_ms, digits = 4))

# View results

M_ms %>% kable(align = c('l', 'c', 'c', 'c'))
