# Supplementary material for: Association of multiple sclerosis with mortality in sepsis: a population-level analysis
Source: J Intensive Care. 2022 Jul 25;10:36. doi: 10.1186/s40560-022-00628-1 (PMC9310428; doi:10.1186/s40560-022-00628-1)
Supplement: Supplementary file 5 — Additional file 5: Multilevel logistic regression for the association of multiple sclerosis with short-term mortality for the whole cohort: alternative modeling for the impact of missing gender data. [file 40560_2022_628_MOESM5_ESM.docx]

| **eTable 4. Multilevel~~, mixed-effects~~ logistic regression for the association of multiple sclerosis with short-term** | | |
| --- | --- | --- |
| **mortality for the whole cohort: alternative modeling for the impact of missing gender data** | |  |
|  |  |  |
| **Modeling approach** | **Adjusted odds ratio (95% CI)^a^** | ***p* value** |
| Include only hospitalizations with gender data | 0.828 (0.723-0.947) | 0.0061 |
| Include hospitalizations with missing gender data as indicator variable | 0.847 (0.742-0.967) | 0.0142 |
| a 95% CI: 95% confidence intervals |  |  |
