# Supplementary material for: Association of multiple sclerosis with mortality in sepsis: a population-level analysis
Source: J Intensive Care. 2022 Jul 25;10:36. doi: 10.1186/s40560-022-00628-1 (PMC9310428; doi:10.1186/s40560-022-00628-1)
Supplement: Supplementary file 7 — Additional file 7: Multilevel logistic regression for the association of multiple sclerosis with short-term mortality among hospitalizations admitted to ICU: alternative modeling for the impact of missing gender data. [file 40560_2022_628_MOESM7_ESM.docx]

| **eTable 6. Multilevel~~,~~ mixed-effects logistic regression for the association of multiple sclerosis with short-term** | | |
| --- | --- | --- |
| **mortality among ICU admissions: alternative modeling for the impact of missing gender data** | |  |
|  |  |  |
| **Modeling approach** | **Adjusted odds ratio (95% CI)^a^** | ***p* value** |
| Include only hospitalizations with gender data | 0.914 (0.759-1.101) | 0.3457 |
| Include hospitalizations with missing gender data as indicator variable | 0.937 (0.780-1.124) | 0.4847 |
| a 95% CI: 95% confidence intervals | | |
